# The Use of Mobile Health Care Among Medical Professionals in the Sichuan-Chongqing Region: Cross-Sectional Survey Study

**DOI:** 10.2196/59153

**Published:** 2024-10-24

**Authors:** Yan Tang, Juan Yang, Ni Wang, Xin Wang, Wenli Hu

**Affiliations:** 1 Department of Neurology, Chongqing Red Cross Hospital (People’s Hospital of Jiangbei District), Chongqing, China; 2 Department of Neurology, The Third Affiliated Hospital of Chongqing Medical University, No. 1 Shuanghu Branch Road, Huixing Street, Yubei District, Chongqing, 401120, China, 86 15823847986; 3 Department of Neurology, JiuLongPo Distict People 's Hospital, Chongqing, China

**Keywords:** health care professionals, mobile health care, technical training, cross-sectional survey, utilization, mobile, usage, China, web-based questionnaire, logistic regression, training, support

## Abstract

**Background:**

The emergence and integration of mobile health care technology have fundamentally transformed the health care industry, providing unprecedented opportunities to improve health care services and professional practice. Despite its immense potential, the adoption of mobile health care technology among health care professionals remains uneven, particularly in resource-limited regions.

**Objective:**

This study aims to explore the use and influencing factors of mobile health care among health care professionals in the Sichuan-Chongqing region of China and make recommendations.

**Methods:**

Convenience sampling was used in a cross-sectional study conducted from November 8 to November 14, 2023, to survey frontline clinical health care professionals at 5 district-level secondary public hospitals in the Sichuan-Chongqing region. A web-based questionnaire was used to investigate the use of mobile health care and its influencing factors among the participants. Descriptive analysis and logistic regression analysis were used in the study.

**Results:**

A total of 550 valid questionnaires were completed. Among the surveyed health care professionals, only 18.7% (103/550) used mobile health care, with a satisfaction rate of only 50.5% (52/103). Around 81.3% (447/550) did not use any form of mobile health care. The age group of 30‐39 years was found to be a significant factor influencing the use of mobile health care by health care professionals (*P*=.03). The main reasons for not using mobile health care among health care professionals were lack of appropriate technical training and support (266/447, 59.5%), lack of suitable management-specific apps (204/447, 45.6%), and concerns about increased workload (180/447, 40.3%). There were significant differences in the single-factor analysis of the reasons for the nonuse of mobile health care among health care professionals from different specialties (*P*=.04). Logistic regression analysis indicated that age was the only significant factor influencing the use of mobile health care by health care professionals (*P*=.04).

**Conclusions:**

The utilization rate of mobile health care among health care professionals in the Sichuan-Chongqing region is low. Age is a significant factor that influences whether health care professionals use mobile health care. Providing appropriate technical training and support may help improve the enthusiasm of health care professionals in using mobile health care.

## Introduction

Currently, with the rapid development of internet technology both domestically and internationally, mobile health, mobile internet, health management, and medical system informatization have become hot research topics in China in recent years [1]. Internet health care refers to the integration of medical information and interconnectedness, encompassing various forms of health care services such as medical information, electronic health records, disease risk assessment, web-based disease consultation, telemedicine, and rehabilitation. Mobile health care, on the other hand, combines internet health care with mobile apps, focusing on medical and health-related apps. As a new health care model actively encouraged by the government, mobile health care can better meet the increasing health care demands of the people and contribute to the realization of the “Healthy China” strategic goals.

In recent years, the aging process of the population in China has been accelerating, leading to an increase in the number of chronic disease patients. The application of mobile health care technology in the field of patient management is increasingly showing its key role, especially in improving work efficiency and patient satisfaction [2]. Traditional health care models face challenges due to factors like geography, economy, transportation, and facilities, complicating patient education and disease management for health care professionals. In contrast, mobile health care eliminates these barriers, enabling health care professionals to continuously track patient conditions throughout the entire disease management cycle, thus demonstrating its wide applicability and significant promotional value. In addition, the application of mobile health care technology not only enhances the accessibility and continuity of health care services but also provides patients with more personalized and convenient medical experiences, further promoting the innovation and progress of health care service models. However, in China, especially in rural areas, the grassroots medical and health information network is still not well established. Large level-III hospitals focus on the diagnosis and treatment of difficult and critical cases, leaving little energy for patient follow-up management. Therefore, we believe that using mobile health care for patient management deserves more attention and implementation from secondary general hospitals.

Previous research on mobile health care in China has primarily focused on the construction of telemedicine platforms and the development and application of mobile health care information systems [3,4], or on patient use patterns [5-7]. However, it has largely overlooked the fact that, within the realm of mobile health care, the acceptance and use by health care professionals are crucial determinants of patient engagement. Therefore, investigating both the adoption patterns and the factors influencing health care professionals’ use of mobile health care technologies is essential.

From the perspective of the growth rate of the mobile health care market in China, the national growth rate has slowed down, and users in major cities have gradually become accustomed to using mobile health care. In contrast, in the southwestern region, particularly the Sichuan-Chongqing area—a key medical hub—the use of mobile health care by professionals not only affects the local service quality but also impacts the broader regional health care landscape. Despite this importance, studies focusing on this demographic within the region are absent. To address this gap, this study surveys the current use and influencing factors of mobile health care among health care professionals in the Sichuan-Chongqing area and provides recommendations. This study is not just about mapping the current use of these technologies. It is about understanding the factors that encourage or deter health care professionals from adopting mobile health tools in their daily practice. By focusing on this underexplored area, our findings aim to inform more than just policy—it is about enabling better health outcomes and shaping a future where health care reaches everyone efficiently and effectively in the region.

## Methods

### Study Design and Participants

This was a cross-sectional survey study conducted using convenience sampling. From November 8 to November 14, 2023, a web-based questionnaire survey was conducted among frontline health care professionals. This study investigated 5 district-level secondary public hospitals based on geographic location and population density (million-level population density): 3 located in central urban areas, specifically Chongqing Jiangbei District People’s Hospital, Chongqing Jiulongpo District Second People’s Hospital, and Sichuan Chengdu Chenghua District Third People’s Hospital; and 2 situated in more distant districts, namely Chongqing Shapingba District Chenjiqiao Hospital and Sichuan Anyue County Third People’s Hospital. Prior to the survey, all participants were informed that their participation in this survey was voluntary, and they were informed of the purpose and significance of this survey research. The questionnaire survey was completed anonymously using the platform QuestionStar. All data were anonymized.

### Data Collection

This study used a self-designed questionnaire survey, which included the following aspects: (1) general information about the participants: sex, age, education background, professional title, occupation, and major; (2) whether they use mobile health care; (3) use of mobile health care: the name of the tool used, its functions, and frequency of use; (4) factors influencing the users: work efficiency, patient compliance, health outcomes, advantages, challenges, satisfaction, and areas for improvement; and (5) reasons for nonusers: lack of appropriate training and technical support, concerns about patient data privacy and security issues, concerns about increased workload due to web-based apps, lack of suitable apps for specific management purposes, negative or unwilling patient response, and lack of awareness of the potential benefits of web-based apps.

### Statistical Analysis

All data were organized and analyzed using SPSS software (version 18.0; IBM Corporation). Continuous and normally distributed metric data were presented as means and SDs, while nonnormally distributed metric data were presented as medians and IQRs. Categorical data were presented as frequencies and percentages. A chi-square test was used to analyze the correlation between the general characteristics of the participants and the use of mobile health care, as well as the reasons for nonuse. Multivariable logistic regression analysis was conducted to identify the factors influencing the use of mobile health care. A significance level of *P*<.05 was considered statistically significant. The reliability of the self-designed questionnaire was assessed using the Cronbach *α*.

### Ethical Considerations

The study was discussed by the Ethics Committee of Chongqing Red Cross Hospital (People’s Hospital of Jiangbei District), which determined that the research involves the use of human information data but does not cause physical harm, nor does it involve sensitive personal information or commercial interests. Consequently, this study was exempted from ethical review [8].

## Results

### Background Characteristics

The participants in this survey were identified as frontline clinical doctors or nurses, interns, and residents. A small sample questionnaire test was conducted first to assess the reliability of the self-made questionnaire, which showed good reliability (Cronbach *α*=.92). The final formal questionnaire was then determined, and a large sample was collected. A total of 556 health care professionals completed the survey, with 550 valid questionnaires, resulting in a valid response rate of 98.9%. The age of the participants ranged from 18 to 59 years, with an average age of 33.24 (SD 7.02) years. Females accounted for 77.1% (424/550) of the participants. A total of 82% (451/550) of the medical personnel had received undergraduate or higher education, 30.2% (166/550) were nurses, and 64.4% (354/550) were doctors (Table 1).

**Table 1.** Background characteristics of respondents.

Variable	Values
**Sex, n (%)**	
Male	126 (22.9)
Female	424 (77.1)
**Age (years), mean (SD)**	33.2 (7.0)
**Age (years), n (%)**	
<30	171 (31.1)
30‐39	294 (53.5)
>40	85 (15.5)
**Education background, n (%)**	
Junior college or below	99 (18.0)
Undergraduate	280 (50.9)
Postgraduate or above	171 (31.1)
**Professional title, n (%)**	
Junior and below	214 (38.9)
Intermediate	257 (46.7)
Senior	79 (14.4)
**Occupation, n (%)**	
Physician	354 (64.4)
Nurse	166 (30.2)
Technician	18 (3.3)
Others	12 (2.2)
**Major, n (%)**	
Clinical department	445 (80.9)
Ancillary departments	50 (9.1)
Public health and others	55 (10.0)

### Specific Use of Mobile Health Care

According to the survey, only 18.7% (103/550) of health care professionals use mobile health care. The most commonly used platforms include “PDA, Yi Doctor, Yi Nurse, and WeChat,” accounting for 44.7% (46/103) of the total. There are also various other mobile health care platforms such as “Micro Doctor, Good Doctor, 317 Nurse, JD Doctor, Creative Doctor, Doctor’s Palm, Doctor’s Circle,” and so on. The main function used is patient record management, accounting for 78.6% (81/103). The use frequency is at least once a day, accounting for 57.3% (59/103). The majority of respondents believe that using mobile health care can improve work efficiency, improve patient compliance, and enhance patient health. However, the satisfaction rate is only 50.5% (52/103), with the main areas for improvement being data security and training support (Table 2).

**Table 2.** Use of mobile health care.

Use status	Values, n (%)
**Function used**	
Patient record management	81 (78.6)
Appointment management	37 (35.9)
Prescription management	44 (42.7)
Communication and consultation	49 (47.6)
Disease monitoring and tracking	55 (53.4)
Other	13 (12.6)
**Use frequency**	
≥1 time per day	59 (57.3)
≥1 time per week	23 (22.3)
≥1 time per month	6 (5.8)
Occasionally	15 (14.6)
**Impact on work efficiency**	
Improve efficiency	80 (77.7)
No significant impact	17 (16.5)
Decrease efficiency	2 (1.9)
Unclear	4 (3.9)
**Effect on patient compliance**	
Yes	64 (62.1)
No	16 (15.5)
Uncertain	23 (22.3)
**Effect on patient health**	
Yes	67 (65)
No	13 (12.6)
Uncertain	23 (22.3)
**Advantages**	
Improve self-management ability	64 (62.1)
Save time	76 (73.8)
Provide real-time data	76 (73.8)
Increase communication frequency	66 (64.1)
**Challenges**	
Privacy and security	78 (75.7)
Technical barriers	67 (65.0)
Patient response	50 (48.5)
**Satisfaction**	
Satisfied	52 (50.5)
Average	47 (45.6)
Dissatisfied	4 (3.9)
**Areas for improvement**	
Training support	73 (70.9)
Data security	74 (71.8)
User-friendliness	70 (68.0)
Increase functionality	64 (62.1)

### Univariate Analysis for Whether to Use Mobile Health Care

Around 81.3% (447/550) of surveyed health care professionals did not use any mobile health care. Age, particularly the 30‐39 years age group, was found to be a significant factor influencing whether health care professionals use mobile health care (*P*=.03). Other factors such as gender, education level, professional title, occupation, and specialty did not have a significant impact on the use of mobile health care among health care professionals, with no statistically significant differences observed (Table 3).

**Table 3.** Univariate analysis of factors influencing the use of mobile health care among medical personnel.

Factor	Used (n=103)	Not used (n=447)	Chi-square (*df*)	*P* value
**Sex, n (%)**			0.024 (1)	.88
Male	23 (4.2)	103 (18.7)		
Female	80 (14.5)	344 (62.5)		
**Age (years), n (%)**			6.791 (2)	.03
<30	21 (3.8)	150 (27.3)		
30‐39	64 (11.6)	230 (41.8)		
≥40	18 (3.3)	67 (12.2)		
**Education background, n (%)**			4.47 (2)	.11
Junior college or below	16 (2.9)	83 (15.1)		
Undergraduate	62 (11.3)	218 (39.6)		
Postgraduate or above	25 (4.5)	146 (26.5)		
**Professional title, n (%)**			4.067 (2)	.13
Junior and below	32 (5.8)	182 (33.1)		
Intermediate	57 (10.4)	200 (36.4)		
Senior	14 (2.5)	65 (11.8)		
**Occupation, n (%)**			1.73 (2)	.42
Physician	63 (11.5)	291 (52.9)		
Nurse	36 (6.5)	130 (23.6)		
Technician and others	4 (0.7)	26 (4.7)		
**Major, n (%)**			4.638 (2)	.10
Clinical department	90 (16.4)	355 (64.5)		
Ancillary departments	4 (0.7)	46 (8.4)		
Public health and others	9 (1.6)	46 (8.4)		

### Multivariable Logistic Regression Analysis for Whether to Use Mobile Health Care

Multivariable logistic regression analysis was conducted with health care professionals’ use of mobile health care as the dependent variable (1 for use, 0 for nonuse) and gender, age, education, professional title, occupation, and major as independent variables. The results showed that age was the only significant factor influencing the use of mobile health care by health care professionals (*P*=.04) (Table 4).

**Table 4.** Multivariable logistic regression analysis of factors influencing health care professionals’ use of mobile health care.

Independent variables	B^a^	SE	Wald chi-square (*df*)	OR^b^ (95% CI)		*P* value
Sex	0.053	0.272	0.038 (1)	1.054 (0.619‐1.796)		.85
Age	0.5	0.237	4.439 (1)	1.648 (1.035‐2.624)		.04
Education background	−0.278	0.201	1.91 (1)	0.757 (0.511‐1.123)		.17
Professional title	−0.089	0.195	0.207 (1)	0.915 (0.625‐1.340)		.65
Occupation	−0.03	0.191	0.025 (1)	0.971 (0.668‐1.410)		.88
Major	−0.105	0.066	2.482 (1)	0.901 (0.791‐1.026)		.12

a B: regression coefficient.

b OR: odds ratio.

### Analysis of Reasons for Not Using Mobile Health Care

The common reasons identified through the presurvey for healthcare professionals not using mobile health are as follows: lack of appropriate technical training and support (266/447, 59.5%), lack of suitable management for specific applications (204/447, 45.6%), concerns about increased workload (180/447, 40.3%), concerns about patient data privacy and security (165/447, 36.9%), ambiguity regarding the potential benefits of web-based applications (164/447, 36.7%), negative reactions or unwillingness from patients (135/447, 30.2%), and other reasons (74/447, 16.6%).

### Main Reasons for Health Care Professionals Not Using Mobile Health Care

Single-factor analysis of factors influencing health care professionals’ nonuse of mobile health care showed no significant differences in reasons for nonuse based on sex, age, educational background, professional title, and occupation. However, there were certain differences in reasons for nonuse among different majors (*P*=.04). The main reasons for nonuse among clinical department personnel were lack of appropriate technical training and support, lack of suitable management apps for specific types, and concerns about increased workload. The main reasons for nonuse among ancillary department personnel were lack of appropriate technical training and support, lack of awareness of potential benefits of web-based apps, and lack of understanding of potential benefits of web-based apps. The main reasons for nonuse among public health and other department personnel were lack of appropriate technical training and support, lack of suitable management apps for specific types, concerns about patient data privacy and security, and lack of awareness of potential benefits of web-based apps (Table 5).

**Table 5.** Single-factor analysis of reasons for health care professionals’ nonuse of mobile health care.

	Lack of appropriate technical training and support	Concerns about patient data privacy and security	Concerns about increased workload	Lack of suitable management-specific apps	Patients have negative reactions or are unwilling	Not clear about the potential benefits of web-based apps	Others	*P* value
**Sex, n (%)**								.75
Male	60 (58.3)	40 (38.8)	45 (43.7)	44 (42.7)	26 (25.2)	41 (39.8)	13 (12.6)	
Female	206 (59.9)	125 (36.3)	135 (39.2)	160 (46.5)	109 (31.7)	123 (35.8)	61 (17.7)	
**Age, n (%)**								.96
<30	81 (54.0)	52 (34.7)	59 (39.3)	56 (37.3)	42 (28.0)	56 (37.3)	28 (18.7)	
30‐39	143 (62.2)	86 (37.4)	94 (40.9)	117 (50.9)	76 (33.0)	84 (36.5)	37 (16.1)	
≥40	42 (62.7)	27 (40.3)	27 (40.3)	31 (46.3)	17 (25.4)	24 (35.8)	9 (13.4)	
**Education background, n (%)**								.91
Junior college or below	46 (55.4)	31 (37.3)	31 (37.3)	34 (41.0)	28 (33.7)	30 (36.1)	17 (20.5)	
Under graduate	131 (60.1)	82 (37.6)	90 (41.3)	107 (49.1)	66 (30.3)	90 (41.3)	40 (18.3)	
Postgraduate or above	89 (61.0)	52 (35.6)	59 (40.4)	63 (43.2)	41 (28.1)	44 (30.1)	17 (11.6)	
**Professional title, n (%)**								.99
Junior and below	102 (56.0)	69 (37.9)	78 (42.9)	80 (44.0)	52 (28.6)	70 (38.5)	32 (17.6)	
Intermediate	127 (63.5)	72 (36.0)	79 (39.5)	93 (46.5)	66 (33.0)	70 (35.0)	32 (16.0)	
Senior	37 (56.9)	24 (36.9)	23 (35.4)	31 (47.7)	17 (26.2)	24 (36.9)	10 (15.4)	
**Occupation, n (%)**								.09
Physician	173 (59.5)	112 (38.5)	118 (40.5)	139 (47.8)	87 (29.9)	99 (34.0)	41 (14.1)	
Nurse	84 (64.6)	47 (36.2)	56 (43.1)	60 (46.2)	43 (33.1)	55 (42.3)	22 (16.9)	
Technician	9 (34.6)	6 (23.1)	6 (23.1)	5 (19.2)	5 (19.2)	10 (38.5)	11 (42.3)	
**Major, n (%)**								.04
Clinical department	221 (62.3)	132 (37.2)	148 (41.7)	170 (47.9)	114 (32.1)	130 (36.6)	46 (13.0)	
Ancillary departments	19 (41.3)	14 (30.4)	16 (34.8)	17 (37.0)	9 (19.6)	17 (37.0)	15 (32.6)	
Public health and others	26 (56.5)	19 (41.3)	16 (34.8)	17 (37.0)	12 (26.1)	17 (37.0)	13 (28.3)	

## Discussion

### Principal Findings

The global COVID-19 pandemic has greatly increased the use of mobile health care in many countries, especially among populations at high risk, such as those with chronic underlying conditions [9]. The demand for mobile health care has surged, but this study found that the use of mobile health care among frontline health care professionals in the Sichuan-Chongqing region is generally poor, with a use rate of only 18.7% (103/550). Similarly, a study by Zhu et al [10] found that only 12.6% of health care professionals have used smart apps. Furthermore, we found that the satisfaction rate among health care professionals who use mobile health care is only 50.5% (52/103), indicating a need for significant improvement in training support and data security. Among nonusers, there are some differences in reasons among different professions, but the main reasons include the lack of appropriate technical training and support, as well as the lack of specific management apps. Therefore, the most prominent issue for surveyed health care professionals, regardless of whether they use mobile health care or not, is the lack of technical training and support.

It is necessary for health care institutions to provide relevant training for health care professionals in using mobile health care. Research has shown that 95% of respondents had not received any training before using mobile health [11], and leadership support and training for health care professionals are crucial in providing mobile health services [12,13]. In this study, the most commonly used mobile health care by health care professionals were “PDA, Yi Doctor, Yi Nurse, and WeChat,” all of which benefited from the unified requirements of the hospital leadership. Other health care professionals also used apps such as “Good Doctor, WeDoctor, 317 Nurse” on their own. A total of 75.7% (78/103) of respondents believed that there are limitations and risks in using personal mobile devices, and the privacy and data security of patients need to be protected. This is similar to the findings of Rowe-Setz et al [14]. Therefore, the popularization of mobile health care requires leadership to lead the development of relevant policies and guidelines, provide practical training for employees, and ensure the smooth and efficient use of technical devices. The content should include specific coverage of the following aspects: (1) basic operations of mobile health care, data security, privacy protection, and how it can help doctors enhance their value and improve their professional skills; (2) provision of real-life cases and user feedback to demonstrate how mobile health care can help health care professionals save time and energy, and enhance job satisfaction; (3) ensuring good integration of mobile apps with existing hospital information systems to avoid duplicate data entry and operational redundancy, thereby reducing workload; (4) establishment of a technical support hotline or web-based platform to promptly address issues and concerns encountered by health care professionals during app use; and (5) in addition to regular training courses for health care professionals, establishment of various guidelines to support mobile health care activities. When mobile health care services are regularly scheduled, they become part of daily work, just like any other familiar operational aspect in the workplace, thereby improving the technical capabilities of professionals. Implementing the above suggestions may help health care professionals overcome barriers to using mobile health care, enhance their willingness and ability to adopt mobile technologies, and promote the digital transformation of health care services and the improvement of health care quality.

Although 81.3% (447/550) of health care professionals surveyed did not use any mobile health services, this does not mean that they lack the willingness to use them. Research has shown that health care professionals have a high acceptance of mobile health services [15]. The lack of use despite the intention is based on the consideration of costs and benefits, which are referred to as perceived benefits. It is also mentioned that building a personal brand for doctors has a positive impact on their willingness to use mobile health care [16,17]. However, there are some health care professionals who hold negative and skeptical attitudes and do not want to acquire the new skills required for mobile health care. Leaders need to try to influence their attitudes and the atmosphere of the workplace to make their use of mobile health care more positive.

The proliferation of mobile health care apps has provided users with more choices, but the wide variety and varying quality of these apps have increased the difficulty for health care professionals to select the right app for managing specific diseases. There have been studies exploring the feasibility of using mobile health care for chronic disease management [18,19]. Therefore, it is important to focus on developing personalized mobile apps tailored to specific medical fields such as hypertension, diabetes, and stroke for effective chronic disease management. The design of mobile health care programs should prioritize the involvement of health care professionals [20], enabling them to better manage patients; promote a shift from doctor-patient relationships to partnerships; and facilitate interdisciplinary team collaboration, patient education, and information sharing across different health care institutions for the same disease. This approach aims to meet the specific needs of health care professionals and achieve the goal of comprehensive management with a patient-centered approach for long-term follow-up care.

Pay attention to seed users and fully tap into the potential user base. Among Chinese mobile phone users, young users account for over 90%. The results of this study show that age, especially the 30‐39 years age group, is a significant factor influencing whether health care professionals use mobile health care. The middle-aged and young user group is large in size, has a strong demand for medical and health services, is more receptive to new things, and has a higher interest in smart apps [21,22]. This suggests that focusing on health care professionals in the 30‐39 years age group as seed users for targeted attention and promotion may achieve more significant results.

### Limitations

In this study, a convenience sampling method was used, limited to health care professionals in secondary public hospitals in the Sichuan-Chongqing region, which may not fully reflect the situation of health care professionals in other regions or hospitals of different levels. In future research, random sampling or stratified sampling methods could be considered to improve the representativeness of the sample. Additionally, efforts should be made to expand the scope of the study to include more regions and different types of hospitals in order to obtain more generalizable research results.

### Conclusion

This survey reveals that frontline health care professionals in the Sichuan-Chongqing region have low use of mobile health care, primarily due to a lack of training support and concerns about data security. Health care professionals have the intention to use it but need to address the cost-benefit issue. It is recommended that leadership provide training support, establish policy guidelines, influence attitudes, and create a positive atmosphere. Emphasis should be placed on the development of personalized applications in the field of chronic disease management, with a focus on the participation of young health care professionals. In summary, efforts should be concentrated on improving training, addressing security concerns, and strengthening leadership guidance to promote the application of mobile health care in the Sichuan-Chongqing region.
